# Pelvic Nephroureterectomy for Renal Cell Carcinoma in an Ectopic Kidney

**DOI:** 10.1155/2012/350916

**Published:** 2012-10-22

**Authors:** Kevin G. Baldie, Usama A. Al-Qassab, Chad W. Ritenour, Muta M. Issa, Adeboye O. Osunkoya, John A. Petros

**Affiliations:** ^1^Atlanta Veterans Affairs Medical Center, 1670 Clairmont Road NE, Decatur, GA 30033, USA; ^2^Department of Urology, Emory University School of Medicine, Clinic B, 1365 Clifton Road, Atlanta, GA 30322, USA; ^3^Department of Pathology and Laboratory Medicine, Emory University, Atlanta, GA 30322, USA; ^4^Department of Hematology and Medical Oncology, Winship Cancer Institute, Emory University, Atlanta, GA 30322, USA

## Abstract

We present a case of an ectopic renal tumor in a 61-year-old morbidly obese man with a pelvic kidney found after presenting with hematuria and irritative voiding symptoms. The mass, along with the ectopic kidney and ureter, was radically resected through an open operation that involved removing both them and the renal vessels from the underlying iliac vessels. Pathological analysis demonstrated an 8.3 cm papillary renal cell carcinoma (RCC) with oncocytic features, Fuhrman nuclear grade 3, with angiolymphatic invasion and negative margins. The patient has been recurrence-free for over four years since tumor resection.

## 1. Introduction

Renal ectopia is a rare condition involving a failure of the mature kidney to reach its normal location within the renal fossa. This congenital anomaly comprises about 0.001% of all autopsies. Pelvic, iliac, abdominal, thoracic, contralateral, and crossed ectopic kidneys can occur. Apart from the development of hydronephrosis and urolithiasis, the ectopic kidney is no more susceptible to disease than the normally positioned kidney [1]. Nevertheless, when considering surgical resection, particularly with the intent to remove a tumor, an accurate understanding of the surrounding anatomy is crucial in order to avoid causing both unnecessary damage to blood vessels and leaving remnants of tumor within the patient.

## 2. Case Report

**Figure 1:**
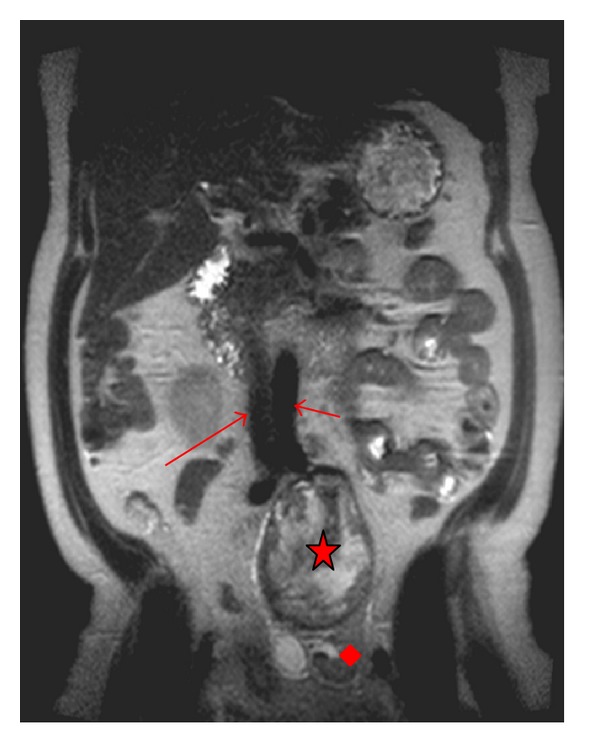
T2-weighted MRI image (coronal view) showing orientation of mass (star) to major vessels. Normal kidney (diamond). Aorta (short arrow). IVC (long arrow). Note that aortic bifurcation occurs at level of superior pole of ectopic left kidney.

**Figure 2:**
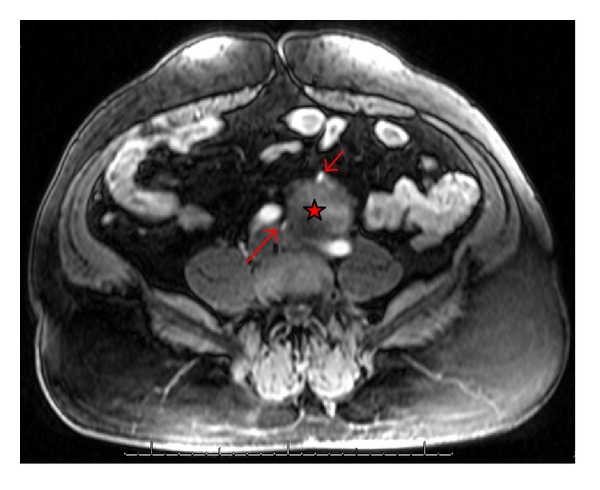
Arterial Phase T1-weighted MRI image (transverse view) showing anomalous arterial supply to ectopic left kidney and mass (star). Proximal left renal artery (short arrow) branching off distal aorta. Distal left renal artery (long arrow) branching off left common iliac artery.

**Figure 3:**
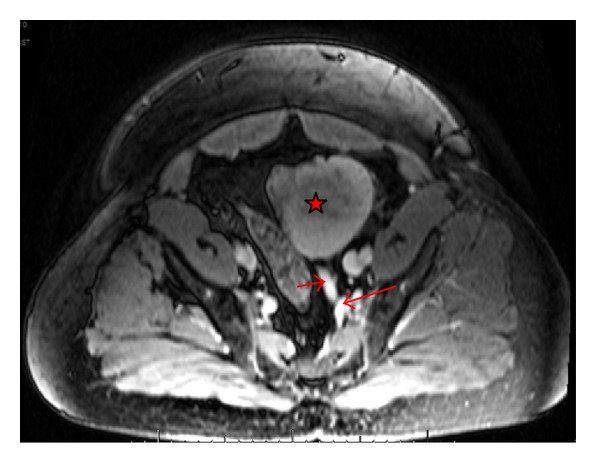
Venous Phase T1-weighted MRI image (transverse view) showing anomalous venous drainage from ectopic left kidney and mass (star). Left renal vein (short arrow) draining into left internal iliac vein (long arrow).

The patient presented as a 61 year old morbidly obese male with a history of chronic kidney disease describing three months of gross, painless hematuria and irritative lower urinary tract symptoms. The remainder of the review of systems was unremarkable. He initially went to an outside hospital after his symptoms were refractory to antibiotic management where he was found on noncontrast CT to have a large left renal mass arising from an ectopic pelvic kidney. Mercaptoacetyltriglycine-3 (MAG3) renography was performed at our institution following referral, showing an essentially nonfunctional left kidney and delayed perfusion, uptake, and excretion in the contralateral kidney. Two weeks later, the patient was further worked up with a complete history and physical, labs, and abdominal MRI with and without contrast. Physical exam and labs were unremarkable except for a creatinine of 2.2. MRI showed an 8.6 cm centrally necrotic, peripherally enhancing solid mass in the superior-interpolar region of the ectopic left kidney worrisome for renal malignancy. The superior margin of the mass rested anterior to the left common iliac vessels (Figure 1). Two anomalous renal arteries supplied the kidney, with the proximal artery branching off of the distal aorta and the distal artery branching of the left common iliac artery (Figure 2). The left renal vein drained into the left internal iliac vein (Figure 3).

**Figure 4:**
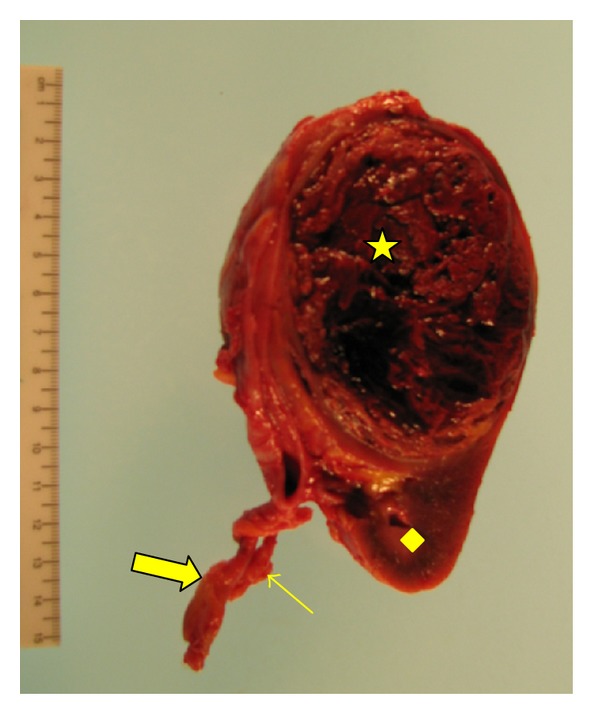
Coronal cut of pelvic/ectopic kidney showing afairly well circumscribed 8.3 × 6.9 × 6.8 cm reddish-brown markedly hemorrhagic mass (star), consistent withpapillary renal cell carcinoma. Other structures include normal renal parenchyma (diamond), ureter (narrow arrow), and left renal artery (thick arrow).

The patient was thoroughly discussed at our institution's weekly genitourinary (GU) preoperative conference with all urology physicians present and, after weighing the risks and benefits, the decision was made for definitive surgical management. The following week, the patient underwent an open transperitoneal radical left pelvic nephroureterectomy. Cystourethroscopy at the onset of the procedure was done to ensure patency of the contralateral ureteral orifice. A midline incision was made from the level of the pubic symphysis to the xiphoid. The space of Retzius was developed bilaterally until there was visualization of the external iliac vessels. After entering the peritoneum and transecting and ligating the proximal and distal urachus, the bladder was dissected down to the level of the vas deferens to aid in visualization. The sigmoid colon was medially displaced and the left colon mobilized medially in order to better access the retroperitoneum. A renal artery was draped over the anterior portion of the kidney, originating from the left common iliac artery. The renal vein took a medial course to reach the left common iliac vein. The kidney was carefully dissected superiorly and inferiorly until the ureter was visualized and tagged. The tumor and kidney were fibrotically adhered to the left common iliac artery, and dissection was done in the midst of confirming palpable lower extremity pulses before and after this stage in the operation. The left renal artery and vein were transected with good hemostasis. The ureter was then clipped distally to the level of the intramural ureter, transected and removed from the pelvis along with the remainder of the specimen (Figure 4) prior to irrigation and layered closure of the peritoneum and abdominal wall.

**Figure 5:**
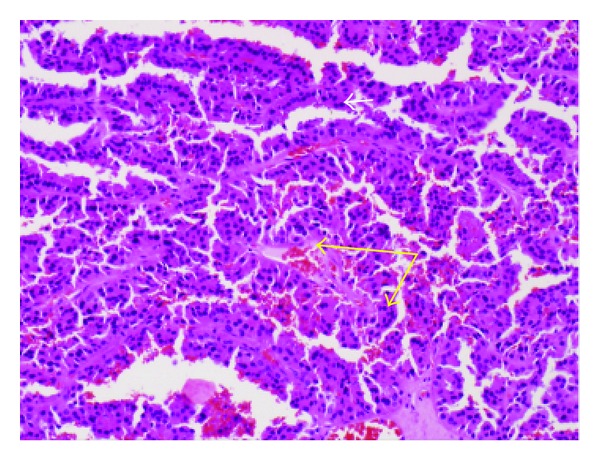
Papillary renal cell carcinoma with oncocytic features (hematoxylin and eosin stain, high magnification). Note distinct papillae with eosinophilic cytoplasm (bottom arrow), with fibrovascular core (top arrow).

 There were no intraoperative complications and the patient tolerated the procedure well. After a low grade fever self-resolved on postoperative day three, the patient progressed as expected and was discharged from the hospital on postoperative day seven shortly following removal of his urethral catheter. Pathology revealed an 8.3 cm papillary renal cell carcinoma (RCC) with oncocytic features, Fuhrman nuclear grade 3, with angiolymphatic invasion and negative ureteral, renal vein, and renal artery margins (Figure 5). The patient was followed up with yearly imaging and, although he was eventually placed on hemodialysis due to end stage renal disease, he has been recurrence-free for over four years since tumor resection.

## 3. Discussion

There is a dearth in the literature discussing renal malignancy in the ectopic pelvic kidney [2, 3]. The advent of improved imaging studies has allowed for a higher incidence in the identification of ectopic kidneys, including those with malignancy. Despite this fact, these patients can still be misdiagnosed, particularly with atypical presentations as in this case. To our knowledge, this is the first case report demonstrating the radical removal of an ectopic pelvic kidney as a result of high grade papillary carcinoma with long term success.

 Ectopic kidney disease is a relatively rare congenital disorder, occurring in about one in 900 autopsies. The incidence of pelvic kidneys is between one in 2100 and one in 3000 autopsies. Embryologically, the uterine bud ascends from the Wolffian duct toward the urogenital ridge by the fourth week of gestation, acquiring a cap of metanephric blastema by the fifth week. Both the uterine bud and developing metanephric blastema migrate cranially and rotate medially along its long axis. This process is normally completed by the eighth week of gestation. Improper migration of the kidney and ipsilateral ureter can result from uterine bud maldevelopment, defective metanephric tissue that fails to induce ascent, genetic abnormalities, and maternal disease or teratogenesis. Ectopic kidney disease can be associated with anomalies of the vertebral column, lower gastrointestinal tract, genital tract, or spinal cord and meninges. Genital abnormalities are the most prominent. For women, these include bicornuate or unicornuate uterus, rudimentary or absent uterus and vagina, and duplication of the vagina. For men, undescended testes, urethral duplication, and hypospadias can occur [1].

The pattern of the renal vascular network is dependent on the position of the ectopic kidney and is completely anomalous. More inferiorly situated ectopic kidneys may be supplied by one or two main renal arteries arising from the distal aorta, aortic bifurcation, and the common or external iliac arteries. The inferior mesenteric arteries can also provide blood supply to these kidneys [1].

 RCC accounts for 80% of all renal malignancies. In 2007, over 51,000 new diagnoses were reported in the United States. According to the 2004 WHO Histological Classification of RCC, clear cell RCC is the most common type, consisting of 75% of all RCC's. This is followed by papillary (10%), chromophobe (5%), hereditary cancer syndromes (5%), and unclassified lesions (4%) [4]. A number of rarer histologic subtypes exists, including multilocular cystic RCC, collecting duct carcinoma, mucinous tubular and spindle cell carcinoma, neuroblastoma-associated RCC, and Xp 11.2 translocation-TFE3 carcinoma, with each making up <1% of RCC [5]. Radiographically, papillary RCC's typically appear hypovascular and homogeneous with limited contrast enhancement on contrast-enhanced CT, although larger tumors may show heterogeneity due to necrosis, hemorrhage, and calcification [1, 5].

 This report describes a case of successful surgical management of a pelvic kidney with cancer. Imaging was carefully analyzed to determine the orientation of the tumor, kidney, renal vessels, and collecting system, and their association with the underlying major vascular structures enabling the surgeons to avoid significant blood loss during dissection of the effected tissue. Negative margins were acquired and close clinical and radiographic follow-up confirmed appropriate surgical technique while helping ensure long-term patient success.
